# Human Peripheral Blood Antibodies with Long HCDR3s Are Established Primarily at Original Recombination Using a Limited Subset of Germline Genes

**DOI:** 10.1371/journal.pone.0036750

**Published:** 2012-05-09

**Authors:** Bryan S. Briney, Jordan R. Willis, James E. Crowe

**Affiliations:** 1 Department of Pathology, Microbiology and Immunology, Vanderbilt University Medical Center, Nashville, Tennessee, United States of America; 2 Department of Pediatrics, Vanderbilt University Medical Center, Nashville, Tennessee, United States of America; 3 Chemical and Physical Biology Program of Vanderbilt University Medical Center, Nashville, Tennessee, United States of America; National Institute of Infectious Diseases, Japan

## Abstract

A number of antibodies that efficiently neutralize microbial targets contain long heavy chain complementarity determining region 3 (HCDR3) loops. For HIV, several of the most broad and potently neutralizing antibodies have exceptionally long HCDR3s. Two broad potently neutralizing HIV-specific antibodies, PG9 and PG16, exhibit secondary structure. Two other long HCDR3 antibodies, 2F5 and 4E10, protect against mucosal challenge with SHIV. Induction of such long HCDR3 antibodies may be critical to the design of an effective vaccine strategy for HIV and other pathogens, however it is unclear at present how to induce such antibodies. Here, we present genetic evidence that human peripheral blood antibodies containing long HCDR3s are not primarily generated by insertions introduced during the somatic hypermutation process. Instead, they are typically formed by processes occurring as part of the original recombination event. Thus, the response of B cells encoding antibodies with long HCDR3s results from selection of unusual clones from the naïve repertoire rather than through accumulation of insertions. These antibodies typically use a small subset of D and J gene segments that are particularly suited to encoding long HCDR3s, resulting in the incorporation of highly conserved genetic elements in the majority of antibody sequences encoding long HCDR3s.

## Introduction

Antibodies containing long heavy chain complementarity determining region 3 (HCDR3) loops have been shown to efficiently neutralize a wide variety of pathogens, including HIV, malaria, and African trypanosomes [1]–[3]. In some cases, the unique feature of long HCDR3 antibodies is that the extended loop structure facilitates interaction with epitopes that are otherwise occult because of extensive glycosylation or location in recessed structures on the pathogen surface. For malaria, antibodies containing long HCDR3s have been identified that bind by extending deep into a hydrophobic cleft on apical membrane antigen 1 (AMA1) to contact highly conserved hydrophobic residues [2]. For HIV, several of the most broad and potently neutralizing antibodies have extremely long HCDR3 loops. Two exceptionally broad and potent anti-HIV antibodies, PG9 and PG16, encode among the longest human antigen-specific antibodies described to date and form secondary structure through the use of a complex hydrogen bonding network in the HCDR3 [4], [5]. These antibodies target a currently undefined quaternary epitope and preferentially bind cell surface expressed trimeric envelope protein [6], [7]. Two additional HIV antibodies, designated 2.5b and 2909, target a similar quaternary epitope and contain long HCDR3s, but are able to neutralize only a very limited panel of virus isolates [8], [9]. A panel of recently described antibodies, PGT141-PGT145, are purported to target the same quaternary epitope as PG9 and PG16, have a similar strong preference for membrane-bound, trimeric envelope, and encode HCDR3s that are even longer than the exceptionally long HCDR3s seen in PG9 and PG16 [10]. The broadly neutralizing HIV antibody b12 contains a long HCDR3 and is able to neutralize by targeting the conserved CD4 binding site [11]–[13]. The b12 antibody uses only heavy chain interactions at the antigen binding interface, and passive administration of b12 has been shown to be protective against low-dose repeated challenge in macaques [14], [15]. Two other broadly neutralizing antibodies with long HCDR3s, 4E10 and 2F5, target a conserved membrane-proximal region and have been shown to protect against mucosal SHIV challenge alone and in combination with the anti-HIV antibody 2G12 [16]–[19], and the long HCDR3 of 2F5 is critical to the neutralizing ability of 2F5 [20]. Antibody 447-52D contains a long HCDR3 loop and is able to neutralize a broad range of clade B HIV-1 isolates by targeting a conserved epitope on the V3 loop of gp120 [21], [22]. Finally, the neutralizing antibody 17b targets the HIV co-receptor binding site and facilitates neutralization by preventing co-receptor binding and reducing affinity for the primary receptor, cluster of differentiation 4 (CD4) [23]. Thus, antibodies containing long HCDR3s comprise a sizeable fraction of the neutralizing HIV antibodies described to date, including many of the most broad and potently neutralizing antibodies.

Although induction of such long HCDR3 antibodies is likely to be critical to the design of an effective HIV vaccine strategy, it is still unclear how to induce such antibodies. Previous work has speculated as to a potential mechanism for inducing such antibodies by vaccination [5], [24]. It is known that the affinity maturation process is associated with codon-length insertion events that are likely caused by the somatic hypermutation machinery [25]–[27]. It is thought, then, that repeated rounds of affinity maturation, resulting in multiple short insertion events within the HCDR3, could gradually lengthen HCDR3 loops in the affinity matured antibodies. This observation fits well with the known kinetics of broadly neutralizing antibody generation during HIV infection: potently neutralizing HIV antibodies are produced later than is common in other viral infections [28], [29], suggesting that many rounds of affinity maturation may be necessary to develop broad and potent neutralizing capacity.

We considered that it is also possible that long HCDR3 loops are not generated primarily through the affinity maturation process, however, and are instead primarily created during the recombination process through the introduction of extensive numbers of N- and P-insertions and the selective use of optimal germline gene segments. If the primary source of long HCDR3 antibodies in the peripheral blood is not affinity maturation, the process of inducing an antibody response containing antibodies with long HCDR3s would consist of exhaustive sampling of the repertoire to select those B cells encoding what are, presumably, rare antibodies [5], [7]. While it has previously been shown that antibodies containing long HCDR3s are present in immature B cell populations in both man [30] and mouse [31], [32] as well as in human perinatal liver [33], it is unclear how frequently these antibodies are able to successfully navigate the autoreactivity screening process and enter the mature B cell population. Extensive work has been done in characterizing short and long HCDR3s in mice [34]–[36], but much of the work was done model systems under non-physiologic conditions. An examination of hundreds of thousands of circulating human antibody sequences has identified an upper limit to the number of unique HCDR3s in a single individual [37]. The upper bound, 3 to 9 million unique HCDR3s per individual, is much lower than previously estimated, but this study did not describe the length distribution of these HCDR3s. It has been shown that many B cells encoding HCDR3s of extreme length are eliminated before reaching the periphery [30], likely because antibodies with long HCDR3s have autoreactive properties [38]–[41]. Detailed study and genetic characterization of the long HCDR3 antibody population in human peripheral blood has been limited by the rarity of such sequences. In this study, we examined expressed antibody sequences from four healthy donors and determined that antibodies containing long HCDR3s are more common in the naïve subset than in memory, indicating that affinity maturation is not the primary source of such antibodies. Further, extensive genetic characterization identified several conserved sequence elements in the long HCDR3 peripheral blood antibody population. Thus, human peripheral blood antibodies containing long HCDR3s are not generated primarily through repetitive rounds of affinity maturation, but are typically formed at the time of the original recombination.

## Results

### Increased HCDR3 Length was not Associated with an Increased Number of Somatic Mutations or Insertions

We considered three major subsets of B cells in the peripheral blood: naïve B cells, which are antigen inexperienced and lack somatic mutations or class-switching; IgM memory B cells, which express the surface memory marker CD27 and show evidence of somatic hypermutation but not class-switching; and IgG memory cells, which express CD27 and have undergone both somatic hypermutation and class-switching. We separately isolated naïve, IgM memory and IgG memory B cells from four healthy individuals (hereafter, designated Group 1) using flow cytometric sorting and subjected the transcribed antibody heavy chain genes from those cells to high throughput sequencing. After selecting only non-redundant, high-quality antibody sequences, we obtained a total of 294,232 naïve cell sequences, 161,313 IgM memory cell sequences and 94,841 IgG memory cell sequences from Group 1 donors. We also subjected the transcribed peripheral blood heavy chain antibody genes of three additional healthy donors (hereafter, designated Group 2) and four HIV-infected donors to high throughput sequencing. The B cells from these additional donors were not sorted by B cell subset prior to sequencing, but instead represent a sampling of the total peripheral blood B cell repertoire from each donor. The number of non-redundant, high-quality sequences obtained from each of the Group 2 and HIV-infected donors is shown in Table S2.

**Figure 1:**
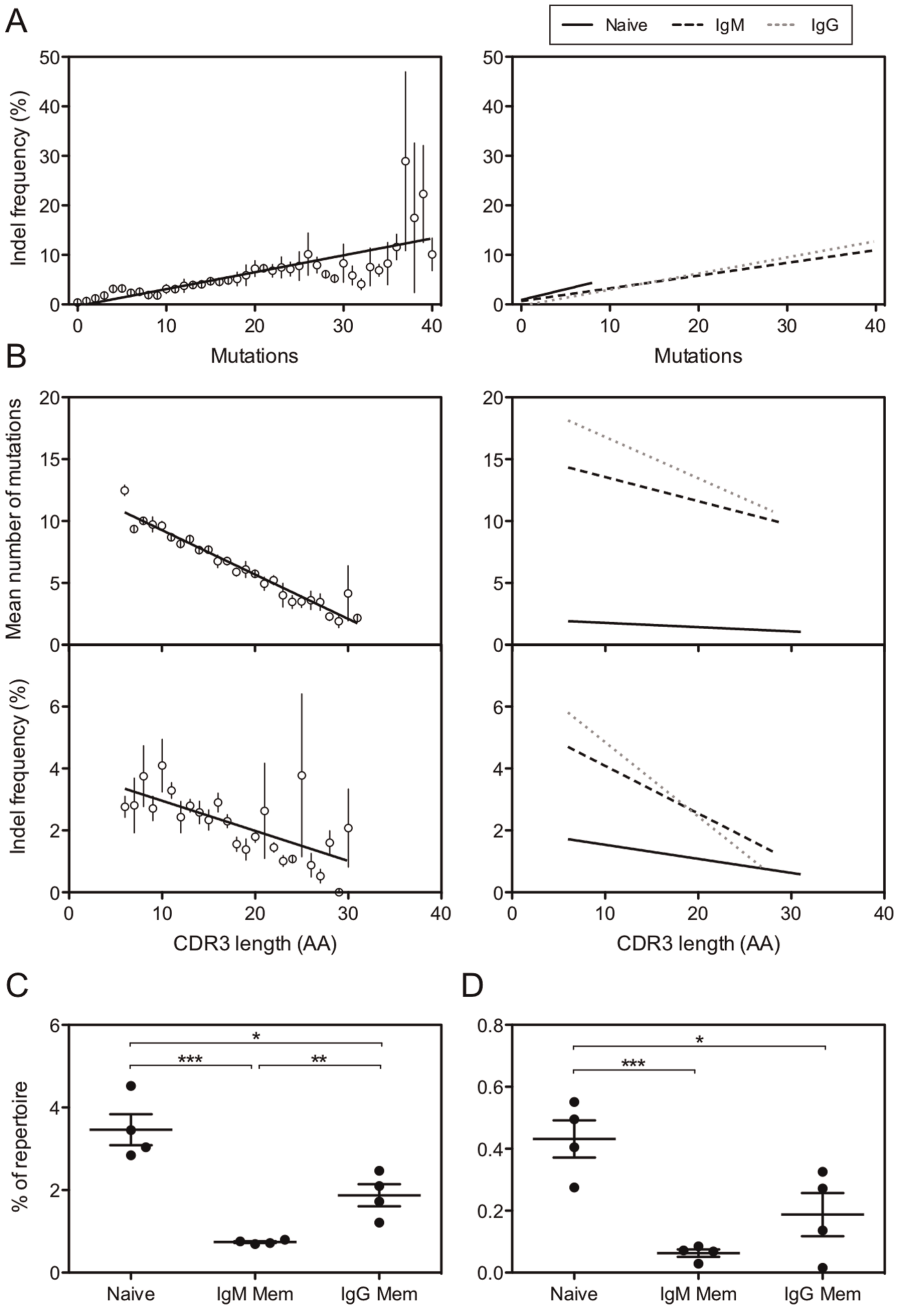
Increased HCDR3 length does not correlate with affinity maturation events. (A) Peripheral blood antibody sequences were grouped by mutation frequency and the percent of sequences in each group that contained codon-length (non-frameshift) insertions was calculated for each donor. All values for healthy donors from Group 1 (n = 4) and Group 2 (n = 3) are shown in the left panel, with the mean percentage ± SEM shown for each mutation value. In the right panel, sequences from Group 1 healthy donors were segregated by B cell subset, and the best-fit linear regression for each subset is shown. (B) Peripheral blood antibody sequences were grouped by HCDR3 length (in amino acids) and the mean number of mutations for each HCDR3 length group was calculated for each donor. As in Figure 1A, the left panel shows the mean ± SEM for all donors in Group 1 and Group 2. The right panel shows the best-fit linear regression of each B cell subset for Group 1 donors. The percent of sequences within each HCDR3 length group containing non-frameshift insertions also was calculated. In the left panel, the mean percentage ± SEM for all donors in Group 1 and Group 2 is shown. In the right panel, the best-fit linear regression of each B cell subset is shown for Group 1 donors. (C) Peripheral blood antibody sequences from Group 1 healthy donors were grouped by donor into naïve and memory subsets and the percent of sequences containing long HCDR3s (≥24 amino acids in length, or two standard deviations above the mean HCDR3 length). The percentages for each donor are shown, with the mean ± SEM. (D) The donor groups from Figure 1C were analyzed for the frequency of very long HCDR3s (≥28 amino acids in length, *i.e.*, three standard deviations above the mean HCDR3 length). The percentages for each donor are shown, with the mean ± SEM. The p values were determined using a one-way ANOVA. All statistically significant differences are indicated. * = p<0.05, ** = p<0.01, *** = p<0.001.

Peripheral blood antibody sequences from Group 1 and Group 2 healthy donors were grouped by mutation frequency, and the fraction of sequences containing insertions was determined for each group (Figure 1A, left panel). In agreement with previously published data [25], we observed a strong positive correlation between number of mutations and insertion frequency (r^2^ = 0.77, p<0.0001). Insertions were present only in a minority of the most highly mutated sequences, however, suggesting either that the somatic hypermutation process is inefficient at introducing insertions in a functional reading frame or that antibodies seldom are able to tolerate genetic insertions while retaining functionality. When separately analyzing each of the B cell subsets from Group 1 healthy donors, we observed that the correlation between mutations and insertions also was found for each of the memory B cell subsets (Figure 1A, right panel).

We next grouped the sequences by HCDR3 length and determined the mean number of mutations and insertion frequency for each HCDR3 length group (Figure 1B, left panels). Interestingly, HCDR3 length was negatively correlated with both mutation frequency (r^2^ = 0.64, p<0.0001) and insertion frequency (r^2^ = 0.13, p<0.0001), suggesting that genetic processes that accomplish somatic hypermutation typically do not alter HCDR3 length. A similar trend was seen in each of the Group 1 healthy donor B cell subsets (Figure 1B, right panels). It has been shown previously that the mean HCDR3 length in circulating memory B cell subsets is shorter than in the naïve B cell subset by approximately a single amino acid [42]. Analyzing the mean HCDR3 length, however, does not allow determination of whether there is a broadly-distributed, overall shortening of the entire HCDR3 repertoire (perhaps caused by somatic hypermuation-induced deletions in the HCDR3 that reduce the length of both long and short HCDR3s) or whether the lower mean HCDR3 length in memory is predominantly due to a strong preference against long HCDR3s in the memory subset. We examined the antibody sequences from naïve, IgM memory, and IgG memory B cell subsets from Group 1 healthy donors for presence of long HCDR3s (defined here as HCDR3s ≥24 amino acids, which corresponds to 2 SD above the mean HCDR3 length) and for very long HCDR3s (defined here as HCDR3s ≥28 amino acids, which corresponds to 3 SD above the mean HCDR3 length). The naïve population contained a significantly higher fraction of long HCDR3s (3.5%, Figure 1C) than both the IgM memory subset (0.74%, p = 0.0003) and the IgG memory subset (1.9%, p = 0.014). The naïve subset also contained a significantly higher fraction of very long HCDR3s (0.43%, Figure 1D) than either the IgM memory subset (0.06%, p = 0.001) or the IgG memory subset (0.19%, p = 0.038). Interestingly, the IgM memory subset showed a significantly reduced frequency of long HCDR3s when compared with the IgG memory subset (p = 0.0056), which supports emerging data that the IgM memory subset does not function primarily as a transition state in progression from the naïve to IgG memory repertoire [42]. In summary, introduction of point mutations and insertions during somatic hypermutation had little effect on HCDR3 length, and we noted the somewhat surprising presence of a large population of long HCDR3s in the naïve repertoire. These data suggested that long HCDR3s are not built primarily through repeated rounds of affinity maturation using genetic insertions, but are present in the naïve repertoire before B cells begin the affinity maturation process.

### Antibody Sequences Encoding Long or Very Long HCDR3s Display Skewed Germline Gene Usage

With the understanding that long HCDR3s are not produced primarily by the affinity maturation process, we examined the antibody repertoire for evidence of recombination events that correlate with HCDR3 length. First, we grouped the peripheral blood antibody sequences from each Group 1 and Group 2 healthy donor by three criteria: (1) all HCDR3s, which included all antibodies containing any HCDR3 length; (2) long HCDR3s, which included only sequences with HCDR3 lengths ≥24 amino acids long; and (3) very long HCDR3s, which included only sequences with HCDR3 lengths ≥28 amino acids long.

**Figure 2:**
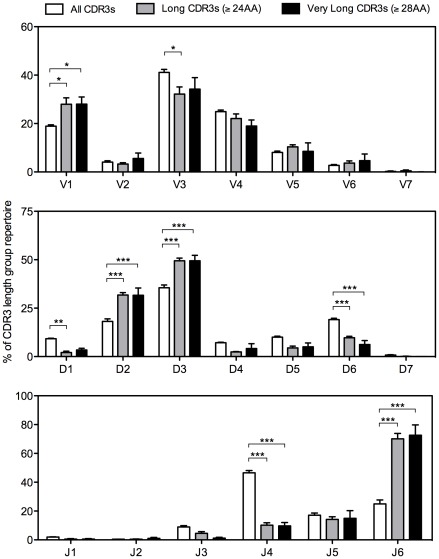
Skewed germline gene usage in antibodies containing long or very long HCDR3s. Peripheral blood antibody sequences from Group 1 (n = 4) and Group 2 (n = 3) healthy donors were assembled into the following three groups by HCDR3 length: (1) all HCDR3s, which contains all sequences of any HCDR3 length; (2) long HCDR3s, which contains only sequences with a HCDR3 length ≥24 amino acids; and (3) very long HCDR3s, which contains only sequences with a HCDR3 length ≥28 amino acids. The frequency of each germline variable gene family, diversity gene family, and joining gene was determined for each HCDR3 length group. The mean frequency ± SEM is shown. All HCDR3 lengths were calculated using the IMGT numbering system. The p values were determined using a two-way ANOVA with Bonferroni post-tests. All statistically significant differences are indicated. * = p<0.05, ** = p<0.01, *** = p<0.001.

We analyzed the germline V, D and J gene segment use in each of the three sequence groups (Figure 2). The most remarkable finding noted was the strong association of two particular D gene families and one J gene segment with longer HCDR3s. Use of D gene families D2 and D3 was increased in long HCDR3s (both D2 and D3: p<0.001) and very long HCDR3s (both D2 and D3: p<0.001). A significant decrease in use of D gene family D6 was seen in long and very long HCDR3s (p<0.01), and a decrease in D1 gene family use was seen in long HCDR3s (p<0.01). Use of J gene J_H_6 was increased markedly in both long (p<0.001) and very long HCDR3s (p<0.001), while joining gene J_H_4 use was decreased in long (p<0.001) and very long HCDR3s (p<0.001). This was not surprising, considering that J_H_6 is the longest J_H_ gene segment and J_H_4 is the shortest. Interestingly, however, use of J_H_1 and J_H_2, which are two amino acids longer than J_H_4, was not increased significantly in long or very long HCDR3 groups. We also noted some variation in V_H_ gene usage. Use of variable gene family V_H_1 was increased in the long HCDR3 (p<0.05) and very long HCDR3 (p<0.05) groups compared to the group with all HCDR3 lengths (p<0.05). V_H_3 family use was decreased in long HCDR3s (p<0.05) but not in the very long HCDR3 group. Separate analysis of the individual B cell subsets in Group 1 donors indicated that the trends observed for the total repertoire were largely mirrored in each subset repertoire (Figures S1,S2,S3).

Deeper analysis of individual D gene use (as opposed to analysis of the D gene families shown in Figure 2) revealed that the increase in D2 and D3 gene families is driven almost completely by increased use of just three of the nine D2 and D3 gene family members: D2-2, D2-15 and D3-3 (Figure S4A). Separate analysis of the frequency of the three D genes that were increased in the total repertoire in each B cell subset (Figure S4B-D) showed similar trends, although the reduced sample size of each subset, as well as the infrequency of long HCDR3s in both memory subsets, resulted in trends that were less robust. The pattern of diversity gene use in long HCDR3s was somewhat surprising, since D3-16, which is not increased in long or very long HCDR3s, is two amino acids longer than any of the three preferred D genes. Further, four additional D genes, D2-8, D3-9, D3-10 and D3-22 are the same length as the three preferred genes, but are not significantly more common in long or very long HCDR3s than in the total repertoire. Thus, while the D2 and D3 gene families encode the longest D genes found in the repertoire, the increased frequency of only a select few of the diversity genes in these families indicates that length is not the only factor driving the increased frequency of these D gene segments in long and very long HCDR3 repertoires.

### Increased HCDR3 Length Correlated with Genetic Features Associated with Recombination

**Figure 3:**
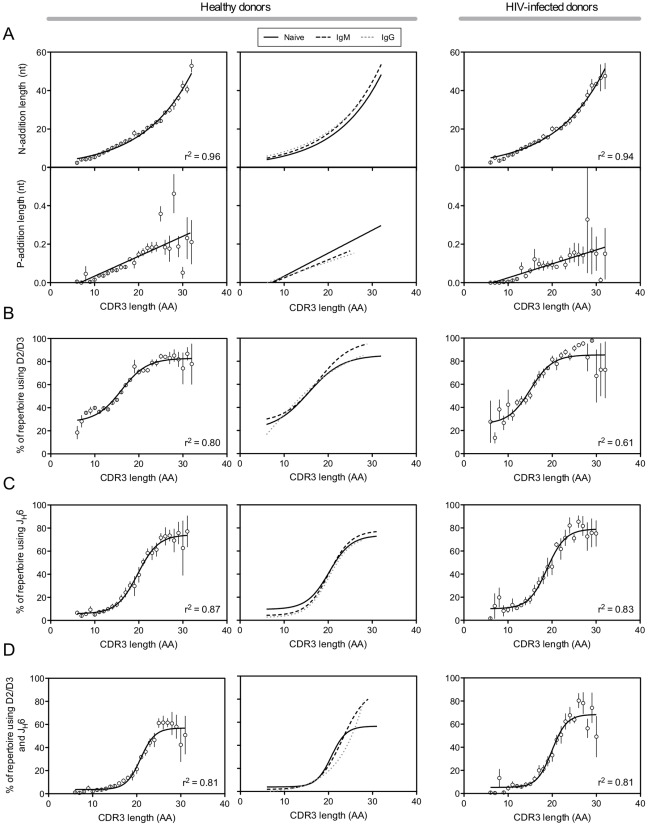
Long HCDR3s correlate with N-addition, P-addition and germline gene usage. For all figure sections, the leftmost panel corresponds to peripheral blood antibody sequences from Group 1 (n = 4) and Group 2 (n = 3) healthy donors. The middle panel corresponds to antibody sequences from Group 1 donors, segregated by B cell subset. The rightmost panel corresponds to peripheral blood antibody sequences from HIV-infected donors (n = 4). (A) Peripheral blood antibody sequences were grouped by HCDR3 length (in amino acids) and the average N-addition length and P-addition length (both in nucleotides) was calculated for each HCDR3 length group. The mean length ± SEM is shown. Regression analysis of N-addition length produced a non-linear, exponential curve of best fit. Regression analysis of P-addition length produced a linear best fit. (B) Peripheral blood antibody sequences were grouped by HCDR3 length and the frequency of sequences encoding either diversity gene family 2 (D2) or diversity gene family 3 (D3) was calculated for each HCDR3 length group. The mean frequency of D2/D3 gene family use ± SEM for each HCDR3 length group is shown. Regression analysis produced a non-linear, sigmoidal curve of best fit. (C) Peripheral blood antibody sequences were grouped by HCDR3 length and the frequency of sequences encoding joining gene 6 (J_H_6) was calculated for each HCDR3 length group. The mean frequency of D2/D3 gene family use ± SEM for each HCDR3 length group is shown. Regression analysis produced a non-linear, sigmoidal curve of best fit. (D) The frequency of sequences encoding both the J_H_6 germline gene and D2/D3 germline gene family members was determined for each HCDR3 length group. The mean frequency of J_H_6/D2/D3 gene family use ± SEM for each HCDR3 length group is shown. Non-linear regression analysis produced a sigmoidal curve of best fit.

We next grouped all of the peripheral blood antibody sequences from Group 1 and Group 2 healthy donors by HCDR3 length and determined the average N-addition length and P-addition length (Figure 3A, left panels) for each HCDR3 length group. Both features showed positive correlations, with N-addition increasing exponentially (r^2^ = 0.96) and P-addition increasing linearly (r^2^ = 0.35, p<0.0001) with increasing HCDR3 length. Similar correlations were seen when analyzing individual B cell subsets from Group 1 healthy donors (Figure 3A, middle panels) and peripheral blood antibody sequences from HIV-infected donors (Figure 3A, right panels).

Following the observation that germline gene usage was skewed in long HCDR3s (Figure 2), we examined the use of D2/D3 (*i.e.*, D2 or D3) family genes as a combined group more closely. We first examined peripheral blood antibody sequences from Group 1 and Group 2 healthy donors (Figure 3B, left panel), and non-linear regression analysis revealed a sigmoidal correlation between D2/D3 gene use (r^2^ = 0.80) and HCDR3 length, with combined D2/D3 gene use exceeding 80% in the longest HCDR3s. The increased use of D2/D3 genes was not surprising, as the D2 and D3 nucleotide sequences are the longest in diversity gene families. This correlation also was seen when analyzing the individual subsets of Group 1 healthy donors (Figure 3B, middle panel) and peripheral blood antibody sequences from HIV-infected donors (Figure 3B, right panel; r^2^ = 0.61). Analysis of J_H_6 gene use in the peripheral blood antibody repertoire of Group 1 and Group 2 healthy donors (Figure 3B, left panel) revealed a sigmoidal correlation between J_H_6 gene use and increasing HCDR3 length (r^2^ = 0.87). J_H_6 was used in less than 10% of the shortest HCDR3s, but was present in over 75% of the longest HCDR3s. We observed a similar correlation when analyzing the individual subsets of Group 1 healthy donors (Figure 3C, middle panel) and peripheral blood antibody sequences from HIV-infected donors (Figure 3C, right panel; r^2^ = 0.83). The observed preference for J_H_6 was expected, as J_H_6 is by far the longest J gene, adding as many as five more codons to the HCDR3 than the shortest J genes. We also examined the peripheral blood repertoire of Group 1 and Group 2 healthy donors to determine the frequency of sequences that use both J_H_6 and D2/D3 genes (Figure 3D, left panel) and found a strong sigmoidal correlation between combined J_H_6 and D2/D3 use (r^2^ = 0.81). While combined J_H_6 and D2/D3 use was unusual in the shortest HCDR3s (3.0%), D2-J_H_6 and D3-J_H_6 encoded antibodies comprised the majority of the repertoire of the longest of HCDR3s (58.8%). The trend toward increased use of J_H_6 and D2/D3 also was seen in individual subsets of Group 1 healthy donors (Figure 3D, middle panel) and in HIV-infected donors (Figure 3D, right panel; r^2^ = 0.81). Further analysis of Group 1 and Group 2 healthy donors revealed a correlation between HCDR3 length and use of J_H_6 in sequences that do not incorporate D2/D3 (Figure S5A) and between HCDR3 length and in use of D2/D3 in sequences that do not incorporate J_H_6 (Figure S5B), but the positive correlation only extended to HCDR3 lengths of approximately 20 amino acids. This finding suggested that while use of one germline gene (either J_H_6 or D2/D3) is sufficient to allow generation of relatively long HCDR3s, both germline genes are required to generate the longest HCDR3s.

### Diversity Gene Reading Frame 2 is Used Preferentially in Long HCDR3s

**Figure 4:**
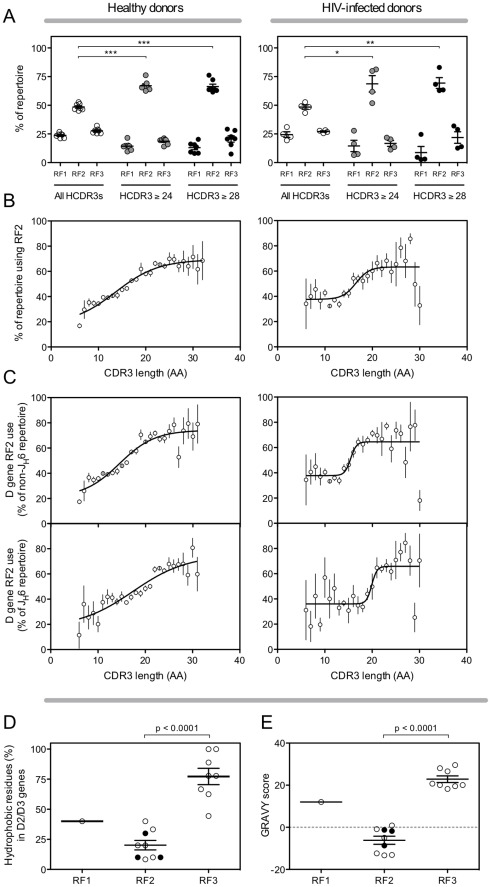
Long HCDR3s preferentially use reading frames (RF) that result in reduced hydrophobicity. (A) Peripheral blood antibody sequences from Group 1 and Group 2 healthy donors (left panel) or HIV-infected donors (right panel) were assembled into three HCDR3 length groups: (1) all HCDR3s; (2) HCDR3s of at least 24 amino acids; and (3) HCDR3s of at least 28 amino acids. (B) The percentage of sequences within each HCDR3 group using reading frame 2 of the diversity gene (RF2) was calculated for each HCDR3 length group. Non-linear regression analysis produced a sigmoidal curve of best fit (r^2^ = 0.84). (C) Sequences that do not encode the joining gene J_H_6 (top panel) or do encode J_H_6 (bottom panel) were grouped by HCDR3 length and RF2 use within each HCDR3 length group was determined. The mean frequency ± SEM is shown. Non-linear regression analysis produced a sigmoidal curve of best fit. (D) The percentage of hydrophobic residues was calculated for each reading frame of every functional (lacking stop codons) diversity gene in the D2 and D3 germline gene families. The mean percentage ± SEM is shown for each reading frame. The RF2 hydrophobicity of the diversity genes which were shown to be increased in long HCDR3s are indicated by filled circles. The p values were determined using a Student’s two-tailed t-test. (E) The grand average of hydropathicity (GRAVY) was calculated for each functional reading frame of each D2 and D3 gene. A positive GRAVY score indicates hydrophobicity, and a negative GRAVY score indicates hydrophilicity. The mean GRAVY score ± SEM is shown for each reading frame. The RF2 GRAVY scores of the diversity genes that were shown to be increased in long HCDR3s are indicated by filled circles. The p values were determined using Student’s two-tailed t-test. All statistically significant differences are indicated. * = p<0.05, ** = p<0.01, *** = p<0.001.

We next analyzed the reading frame preferences in long HCDR3s. We used the ImMunoGeneTics (IMGT) method for calculating the D gene reading frame, which determines the reading frame based on the first codon of the D gene nucleotide sequence. The functional reading frame equivalent to IMGT reading frame 2 (RF2) has been shown to be the most common reading frame in the overall repertoire [30], [31], [34], [36], [43], however, there was a significantly increased preference for RF2 in long HCDR3s in the peripheral blood antibody repertoire of Group 1 and Group 2 healthy donors (Figure 4A, left panel; p<0.001) and in HIV-infected donors (Figure 4A, right panel; p<0.05). A significant increase in RF2 use was also seen in very long HCDR3s in both healthy and HIV-infected donors (Figure 4A; p<0.001 and p<0.01, respectively). Although this reading frame is identified by IMGT as RF2, alternate methods of determining the reading frame, which are based on analysis of the amino acid sequence instead of the nucleotide sequence, produce a different reading frame nomenclature. The reading frame identified as RF2 by IMGT would be identified as RF1 using the alternate “functional” reading frame determination system. To keep confusion to a minimum, the IMGT nomenclature will be used for the remainder of this report. The increased RF2 use in long HCDR3s was unexpected because alternating the reading frame of the D gene should not affect the overall sequence length significantly. However, when analyzing the peripheral blood repertoire of Group 1 and Group 2 healthy donors, we discovered that the frequency of RF2 in the longest HCDR3s (Figure 4B, left panel; 69%) was over twice the frequency of RF2 in the shortest HCDR3s (28%). A similar pattern was seen in HIV-infected individuals (Figure 4B, right panel).

We next considered whether RF2 was selected more frequently in long HCDR3s because use of RF2 may have allowed for more efficient in-frame recombination with the highly preferred J_H_6 gene. Examination of long HCDR3s in Group 1 and Group 2 healthy donors showed a similarly strong preference for RF2 (74%, r^2^ = 0.55) in the longest HCDR3s of recombinants that did not use the J_H_6 gene (Figure 4C, top left panel) than was seen in the total repertoire. The same trend also was seen in sequences that used J_H_6 (Figure 4C, bottom left panel; 75%, r^2^ = 0.44). A similar pattern of RF2 use in the presence or absence of J_H_6 also was seen in cells from the HIV-infected subjects (Figure 4C, right panels). Thus, the observed RF2 preference in long HCDR3s was not primarily due to the need to form in-frame recombinations with J_H_6.

Antibodies with long, hydrophobic HCDR3s often possess autoreactive properties [38]–[41], and RF2 has been shown to be preferred in the antibody repertoire likely due to increased tyrosine frequency and lower hydrophobicity than other reading frames [43], [44]. Diversity genes in the D2 and D3 families are enriched for tyrosine residues, although the three D genes with the highest tyrosine content (D3-10, D3-16 and D3-22) were not among the preferred D genes (data not shown). We next analyzed the frequency of hydrophobic residues (Figure 4D) and the grand average of hydropathicity (GRAVY, Figure 4E) of each functional reading frame for the D2 and D3 families, with special attention paid to the three germline D genes that were found most often in long HCDR3s (designated by filled circles). Although the broader results validate previous data (namely that RF2 is much less hydrophobic than other reading frames), RF2 of the three preferred germline genes is not substantially less hydrophobic than RF2 of the other, non-preferred, D2/D3 gene members. Thus, although hydrophobicity and tyrosine frequency may drive the overwhelming prominence of RF2 in the normal antibody repertoire, they do not seem to account for the strong preference for the three highly preferred D genes in the long HCDR3 repertoire.

### Amino Acid Residues Critical to Binding and Neutralization of HIV by Broadly Neutralizing Antibodies PG9 and PG16 are Encoded by J_H_6 and D3-3 Germline Genes

**Figure 5:**
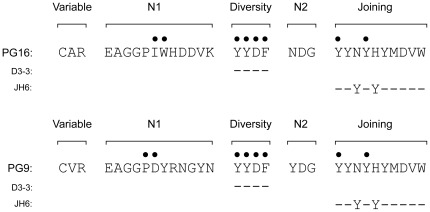
Amino acid residues in J_H_6 and RF2 of D3-3 germline gene segments are critical to binding and neutralization of HIV by long HCDR3-containing antibodies PG9 and PG16. The HCDR3 amino acid sequences of HIV-specific mAbs PG9 and PG16 are shown aligned to the amino acid sequences of germline D3-3 and J_H_6 genes. Dashes in the alignments indicate conservation with the respective PG antibody. Residues shown to be critical to binding or neutralization of HIV, defined as ≥10-fold decrease in either binding or neutralization when mutagenized [5], [24] are indicated by filled circles.

The broadly neutralizing HIV antibodies PG9 and PG16 contain the longest HCDR3s of any antigen-specific human monoclonal antibodies described to date [4]. We considered the genetic basis for development of these unusual antibodies in light of the information presented above on the typical origin of long HCDR3s. These clonally related antibodies both possess HCDR3 regions containing 30 amino acids, which is twice the mean HCDR3 length in the total repertoire. Remarkably, both PG9 and PG16 antibodies use D3-3, one of the three D genes highly preferred in long HCDR3s, and J_H_6. Further, both antibodies position the diversity gene in RF2. We performed amino acid sequence alignments with PG9 or PG16 and the corresponding germline D and J genes (Figure 5). Based on previous studies [7], we identified eight critical residues in these antibodies for which a ≥10-fold decrease in either binding or neutralization occurred when those single amino acids were mutagenized. Interestingly, six of the eight critical residues were encoded by the germline sequence of the D and J genes. Although two additional crucial residues appear to be encoded by N-addition, it is impossible to rule out the possibility that these residues are the consequence of a post-recombination insertion event. Thus, the molecular basis for development of these most broadly neutralizing antibodies for HIV using very long HCDR3s was not a rare occurrence of unusual mutations. Instead, these antibodies were derived from a typical, almost canonical, selection of a D3-3 gene using RF2 and J_H_6, and much of the high affinity of these antibodies derives from interactions mediated by unmutated germline-encoded residues.

## Discussion

Although designing a strategy for selecting or inducing antibodies with long HCDR3s might be a groundbreaking step for vaccine design, very little is known about the genetic origin of such antibodies. Two general models have been proposed for the generation of antibodies with long HCDR3s. First, it is possible that long HCDR3s are generated through the introduction of multiple short insertion events during the somatic hypermutation process [25], [27]. Indeed, we have shown recently that a genetic insertion was a critical feature that mediated affinity maturation and acquisition of neutralizing potency for a human antibody that inhibits influenza virus [45], although this was a short insertion that did not cause a long HCDR3. Alternatively, it is possible that long HCDR3s could be created at the time of recombination through a combination of extensive incorporation of non-templated nucleotides during N- and P-addition and the selective use of longer germline gene segments. In this setting, the extended length of the HCDR3 would be established without the need for the affinity maturation process. In this study, we found that antibodies containing long HCDR3s are created primarily at recombination and not through affinity maturation. In addition, we identified several genetic features that are frequently seen in antibodies with long HCDR3s but are uncommon in the rest of the antibody repertoire. Finally, analysis of long HCDR3 encoding antibody sequences from HIV-infected donors produced results that closely mirrored those from healthy donors. These results suggested that antibodies with long HCDR3s in HIV-infected individuals also typically were generated at the time of recombination.

These data show that increased HCDR3 length does not correlate with either increased mutation count or insertion frequency in the peripheral blood antibody population, although mutation count and insertion frequency are themselves strongly correlated. Further, these studies demonstrate the presence of B cells with receptors incorporating long HCDR3s in the naïve cell population. In fact, the naïve B cell subset contained a higher proportion of long HCDR3s than did the affinity-matured memory B cell population, suggesting that long HCDR3s are produced independent of the affinity maturation process. Together, the lack of correlation between increased HCDR3 length and increased number of insertions arising during affinity maturation and the presence of a sizeable fraction of long HCDR3s in the memory cell population strongly indicate that long HCDR3s are not primarily generated by repeated rounds of affinity maturation.

We also examined several events that occur during the recombination process and discovered many correlations between these molecular features of recombination and increased HCDR3 length. First, we found that increased number of nucleotides introduced by N- or P-addition both correlated with increased HCDR3 length. Next, we found that germline diversity genes in the D2/D3 family and the germline joining gene J_H_6 were highly favored in long HCDR3s. In fact, over half of all of the longest HCDR3s used both of these germline sequence elements, while fewer than 5% of the shortest HCDR3s contained both sequence elements. Finally we discovered, somewhat surprisingly, that RF2 was highly preferred in long HCDR3s. Previous work by several groups has indicated that long, hydrophobic HCDR3s often possess autoreactive properties [38]–[41], so it seems likely that RF2 is used preferentially primarily because of the reduced hydrophobicity profile compared to other reading frames.

The observation that long HCDR3s are composed of conserved sequence elements is of critical importance for two reasons. First, these findings provide information that might be used in the design of strategies to selectively induce expansion of particular B cells encoding antibodies with long HCDR3s. Since long HCDR3s are generated using a limited set of germline gene segments, and since those germline segments are rarely used in short HCDR3s, immunogens designed to target these conserved sequence elements might induce an antibody response that is enriched in antibodies containing long HCDR3s. Second, knowledge of conserved genetic elements present in the majority of long HCDR3s provides a starting point for affinity maturation of these antibodies. For example, it has been suggested that one route to development of an HIV vaccine would be to identify structures of neutralizing epitopes and design immunogens that mimic these epitopes, with the goal of eliciting an antibody response focused on the desired neutralizing epitope [46]–[49]. An alternative to this approach has been proposed, however, which consists of identifying the structural and genetic components of potently neutralizing antibodies and designing immunogens that gradually and specifically induce desired affinity maturation events that result production of broadly neutralizing antibodies. In effect, this alternative process would involve rationally guiding the affinity maturation process through the selective use of sequential immunizations [5]. This strategy would require detailed knowledge not only of the desired final product, in this case a PG9- or PG16-like broadly neutralizing antibody, but also of the genetic characteristics of the naïve predecessors of the desired broadly neutralizing antibody. The work presented here provides a substantial step toward realization of this alternative method of vaccine development. Until this study, little was known about the process of generating antibodies with long HCDR3s or the genetic characteristics of the naïve predecessors of such antibodies. We have identified conserved genetic elements in the long HCDR3 antibody population that form a potential starting point from which rationally guided affinity maturation may begin.

In the case of mAbs PG9 and PG16, this potential is especially enticing. Since many of the residues in these mAbs that are critical for binding and neutralization are encoded in the germline gene sequence, little affinity maturation may be necessary to produce a potently neutralizing antibody. In fact, all but two residues identified in PG9 and PG16 as critical to binding and neutralization were present in the germline D3-3 or J_H_6 genes, and those additional critical residues were generated by random N-addition. Accordingly, we suggest that it is highly likely that there are naïve antibodies in HIV-unexposed individuals that use D2/3 and J6 gene segments and by random happenstance of N-addition, encode many of the residues critical to PG-like neutralization. Thus, while germline reversions of PG9 and PG16 are non-neutralizing [5], it is possible that the naive predecessors of PG-like antibodies will require only limited affinity maturation to gain neutralization capacity. If this is the case, efforts should be focused on selective induction of these rare antibodies containing long HCDR3s, rather than sequential immunization strategies to “build up” long HCDR3s with somatic insertions.

In summary, we have identified the primary genetic basis of development of antibodies containing long HCDR3s. These antibodies are present in naïve populations, indicating that affinity maturation is not necessary to produce such antibodies. Further, we have identified conserved genetic features of such antibodies, providing a potential pathway for selective induction of antibodies containing long HCDR3s.

## Methods

### Sample Preparation and Sorting

Peripheral blood was obtained from healthy adult donors following informed consent, under a protocol approved by the Vanderbilt Institutional Review Board. Mononuclear cells from the blood of four donors were isolated by density gradient centrifugation with Histopaque 1077 (Sigma). Prior to staining, B cells were enriched by paramagnetic separation using microbeads conjugated with antibodies to CD19 (Miltenyi Biotec). Enriched B cells were washed twice with PBS/5% FBS and stained with the following antibodies conjugated to fluorophores: CD19-PerCP-Cy5.5, CD27-APC, IgM-PE, IgG-FITC, CD14-Horizon V450 and CD3-Horizon V450 (Becton Dickinson). Cells from particular B cell subsets were sorted as separate populations on a high speed sorting cytometer (FACSAria III; Becton Dickinson) using the following phenotypic markers, naïve B cells: CD19^+^/CD27^−^/IgM^+^/IgG^−^/CD14^−^/CD3^−^, IgM memory B cells: CD19^+^/CD27^+^/IgM^+^/IgG^−^/CD14^−^/CD3^−^ and IgG memory B cells: CD19^+^/CD27^+^/IgM^−^/IgG^+^/CD14^−^/CD3^−^. Following flow cytometric sorting, total RNA was isolated from each sorted cell subset using a commercial RNA extraction kit (RNeasy; Qiagen) and stored at −80**°**C until analysis.

### cDNA Synthesis and PCR Amplication of Antibody Genes

RT-PCR primers were originally described by the BIOMED-2 consortium [50] and were slightly modified to suit amplification for large-scale parallel pyrosequencing (454 Sequencing; 454 Life Sciences/Roche). 100 ng of each total RNA sample and 10 pmol of each RT-PCR primer (Table S1) were used in duplicate 50 µl RT-PCR reactions using the OneStep RT-PCR system (Qiagen). Thermal cycling was performed in a BioRad DNA Engine PTC-0200 thermal cycler using the following protocol: 50°C for 30:00, 95°C for 15:00, 35 cycles of (94°C for 0:45, 58°C for 0:45, 72°C for 2:00), 72°C for 10:00. cDNA synthesis and amplification were verified by agarose gel electrophoresis before duplicate RT-PCR reactions were pooled. 5 µl of each pooled RT-PCR reaction was used as template for 100 µl 454-adapter PCR reactions, which were carried out in quadruplicate. 20 pmol of each 454-adapter primer (Table S1) and 0.25 units of AmpliTaq Gold polymerase (Applied Biosystems) were used for each reaction. Thermal cycling was performed in a BioRad DNA Engine PTC-0200 thermal cycler using the following protocol: 95°C for 10:00, 10 cycles of (95°C for 0:30, 58°C for 0:45, 72°C for 2:00), 72°C for 10:00. Amplification was verified by agarose gel electrophoresis before quadruplicate 454-adapter PCR reactions were pooled.

### Amplicon Purification and Quantification

Amplicons were purified from the pooled 454-adapter PCR reactions using the Agencourt AMPure XP system (Beckman Coulter Genomics). Purified amplicons were analyzed on a 2% agarose gel to verify complete primer removal before quantification using a Qubit fluorometer (Invitrogen).

### Amplicon Nucleotide Sequence Analysis

The size and quality of the DNA libraries was evaluated on an Agilent Bioanalyzer 2100 using the DNA 7500 Labchip (Agilent). The samples then were diluted to a working concentration of 1×10^6^ molecules per µL. Quality control of the amplicon libraries and emulsion-based clonal amplification and sequencing on the 454 Genome Sequencer FLX Titanium system were performed by the W. M. Keck Center for Comparative and Functional Genomics at the University of Illinois at Urbana-Champaign, according to the manufacturer’s instructions (454 Life Sciences). Signal processing and base calling were performed using the bundled 454 Data Analysis Software version 2.5.3 for amplicons.

### Antibody Sequence Analysis

Nucleotide sequences were compared to the reference sequences from IMGT, the international ImMunoGeneTics information system (http://www.imgt.org) [51] and analyzed using IMGT/HighV-QUEST, a web portal allowing the analysis of thousands of sequences on IMGT/V-QUEST [52]. IMGT output was parsed into a custom MySQL database for further analysis. Database administration and querying were performed with Navicat for MySQL (Navicat). Antibody sequences returned from IMGT were considered to be “high-quality” antibody sequences if they met the following requirements: sequence length of at least 300 nt; identified variable and joining genes; an intact, in-frame recombination; and absence of stop codons or ambiguous nucleotide calls within the reading frame. HCDR3 length was determined using the IMGT numbering system.

### Statistical Analysis

All statistical analysis was performed using Graphpad Prism software.

## Supporting Information

Figure S1 **Skewed germline gene usage in naïve antibodies containing long or very long HCDR3s.** Peripheral blood antibody sequences from the naïve subset of Group 1 (n = 4) healthy donors were assembled into the following three groups by HCDR3 length: (1) all HCDR3s, which contains all sequences of any HCDR3 length; (2) long HCDR3s, which contains only sequences with a HCDR3 length ≥24 amino acids; and (3) very long HCDR3s, which contains only sequences with a HCDR3 length ≥28 amino acids. The frequency of each germline variable gene family, diversity gene family, and joining gene was determined for each HCDR3 length group. The mean frequency ± SEM is shown. All HCDR3 lengths were calculated using the IMGT numbering system. The p values were determined using a two-way ANOVA with Bonferroni post-tests. All statistically significant differences are indicated. * = p<0.05, ** = p<0.01, *** = p<0.001.
(TIF)

Figure S2 **Skewed germline gene usage in IgM memory antibodies containing long or very long HCDR3s.** Peripheral blood antibody sequences from the IgM memory subset of Group 1 (n = 4) healthy donors were assembled into the following three groups by HCDR3 length: (1) all HCDR3s, which contains all sequences of any HCDR3 length; (2) long HCDR3s, which contains only sequences with a HCDR3 length ≥24 amino acids; and (3) very long HCDR3s, which contains only sequences with a HCDR3 length ≥28 amino acids. The frequency of each germline variable gene family, diversity gene family, and joining gene was determined for each HCDR3 length group. The mean frequency ± SEM is shown. All HCDR3 lengths were calculated using the IMGT numbering system. The p values were determined using a two-way ANOVA with Bonferroni post-tests. All statistically significant differences are indicated. * = p<0.05, ** = p<0.01, *** = p<0.001.
(TIF)

Figure S3 **Skewed germline gene usage in IgG memory antibodies containing long or very long HCDR3s.** Peripheral blood antibody sequences from the IgG memory subset of Group 1 (n = 4) healthy donors were assembled into the following three groups by HCDR3 length: (1) all HCDR3s, which contains all sequences of any HCDR3 length; (2) long HCDR3s, which contains only sequences with a HCDR3 length ≥24 amino acids; and (3) very long HCDR3s, which contains only sequences with a HCDR3 length ≥28 amino acids. The frequency of each germline variable gene family, diversity gene family, and joining gene was determined for each HCDR3 length group. The mean frequency ± SEM is shown. All HCDR3 lengths were calculated using the IMGT numbering system. The p values were determined using a two-way ANOVA with Bonferroni post-tests. All statistically significant differences are indicated. * = p<0.05, ** = p<0.01, *** = p<0.001.
(TIF)

Figure S4 **Frequency of D gene use in long and very long HCDR3s.** Antibody sequences from Group 1 healthy donors (n = 4) were assembled into the following three groups by HCDR3 length: (1) all HCDR3s, which contains all sequences of any HCDR3 length; (2) long HCDR3s, which contains only sequences with a HCDR3 length ≥24 amino acids (at least two standard deviations above the mean HCDR3 length for the entire repertoire); or (3) very long HCDR3s, which contains only sequences with a HCDR3 length ≥28 amino acids (at least three standard deviations above the mean). The frequency of diversity gene use in each of the three groups was determined for Group 1 healthy donor sequences (A). For the diversity genes that showed significant increases in (A), frequency was calculated for the naive (B), IgM memory (C), and IgG memory (D) subsets from the same Group 1 donors. The mean frequency ± SEM is shown. All HCDR3 lengths were calculated using the IMGT numbering system. The p values were determined using a two-way ANOVA with Bonferroni post tests. All statistically significant differences are indicated. * = p<0.05, ** = p<0.01, *** = p<0.001.
(TIF)

Figure S5 **Limited preference for isolated use of D2/D3 or J_H_6 in the longest HCDR3s.** Peripheral blood antibody sequences from Group 1 (n = 4) and Group 2 (n = 3) healthy donors were grouped by HCDR3 length (in amino acids) and the frequency of (A) sequences encoding germline genes from the D2 or D3 families but not J_H_6 or (B) sequences encoding J_H_6 but not either of the D2 or D3 families. The mean frequency ± SEM is shown. All HCDR3 lengths were calculated using the IMGT numbering system.
(TIF)

Table S1 **Primers used in RT-PCR and 454-Adapter PCR.** Multiplex identifiers (MIDs) are underlined. Forward primers use MID7 and reverse primers use MID4 (Roche). Additional complementarity between RT-PCR primers and 454-Adapter PCR primers is identified in italics. All primer sequences are in the 5′ to 3′ orientation.
(DOCX)

Table S2 **Sequencing results from Group 2 healthy donors and HIV-infected donors.** Donor IDs with the format 100XX are HIV-infected donors. Donors with the format HD-XXX and Subject 42 are Group 2 healthy donors. The number of sequences includes only non-redundant, high-quality sequences.
(DOCX)
