# Supplementary figures and images for: Human Peripheral Blood Antibodies with Long HCDR3s Are Established Primarily at Original Recombination Using a Limited Subset of Germline Genes
Source: PLoS One. 2012 May 9;7(5):e36750. doi: 10.1371/journal.pone.0036750 (PMC3348910; doi:10.1371/journal.pone.0036750)

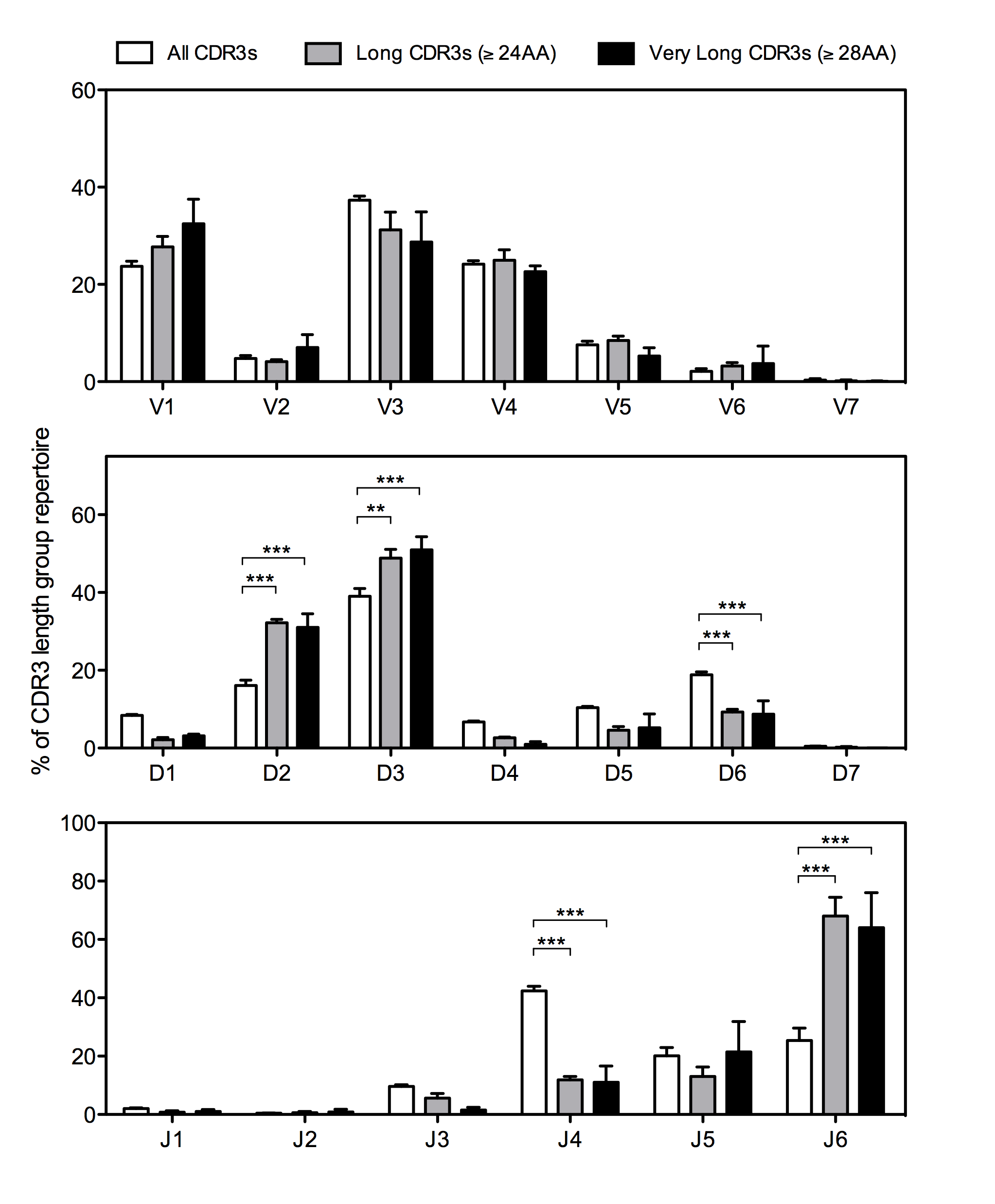

Supplement: Figure S1 — Skewed germline gene usage in naïve antibodies containing long or very long HCDR3s. Peripheral blood antibody sequences from the naïve subset of Group 1 (n = 4) healthy donors were assembled into the following three groups by HCDR3 length: (1) all HCDR3s, which contains all sequences of any HCDR3 length; (2) long HCDR3s, which contains only sequences with a HCDR3 length ≥24 amino acids; and (3) very long HCDR3s, which contains only sequences with a HCDR3 length ≥28 amino acids. The frequency of each germline variable gene family, diversity gene family, and joining gene was determined for each HCDR3 length group. The mean frequency ± SEM is shown. All HCDR3 lengths were calculated using the IMGT numbering system. The p values were determined using a two-way ANOVA with Bonferroni post-tests. All statistically significant differences are indicated. * = p<0.05, ** = p<0.01, *** = p<0.001. (TIF) [file pone.0036750.s001.tif]

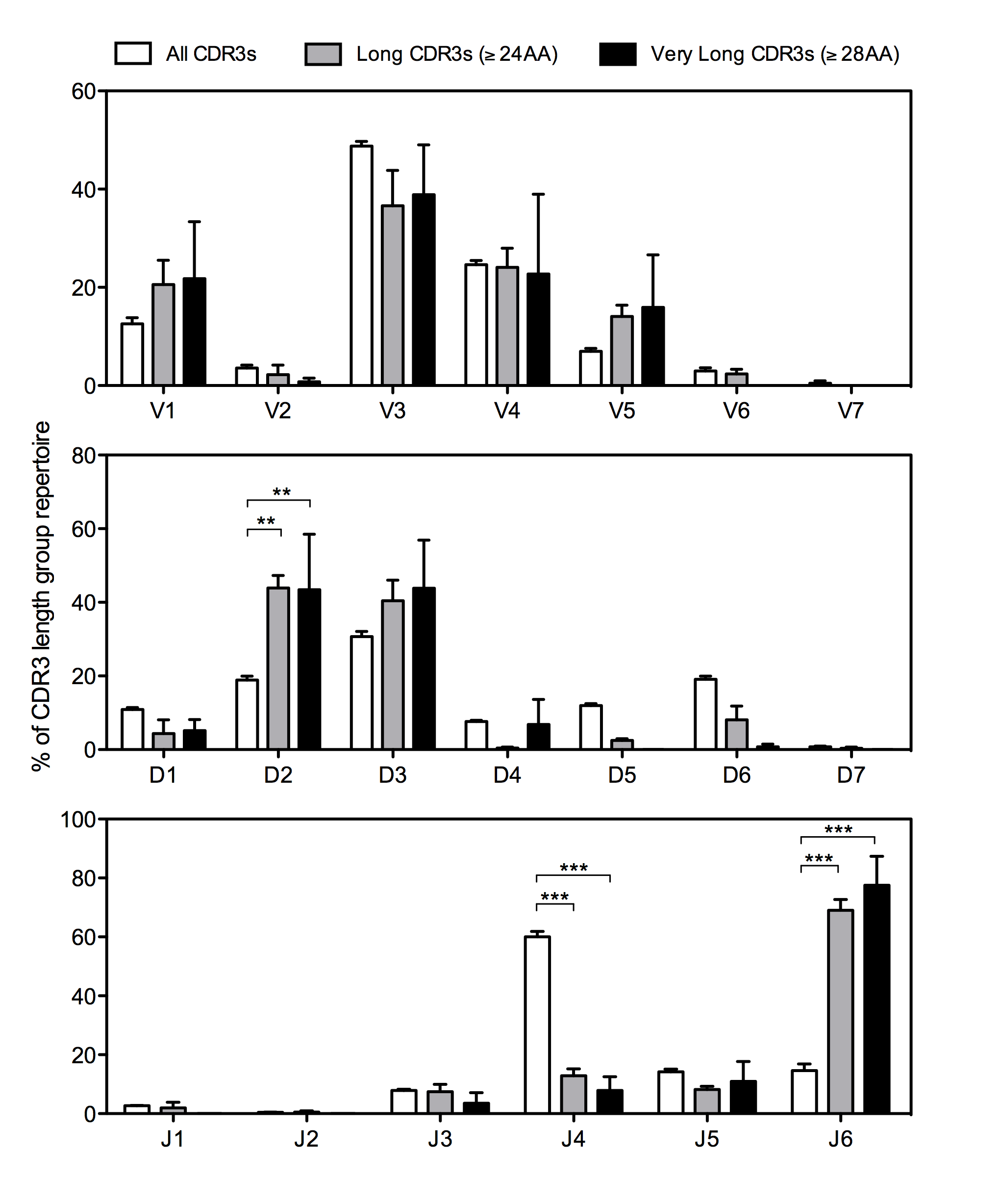

Supplement: Figure S2 — Skewed germline gene usage in IgM memory antibodies containing long or very long HCDR3s. Peripheral blood antibody sequences from the IgM memory subset of Group 1 (n = 4) healthy donors were assembled into the following three groups by HCDR3 length: (1) all HCDR3s, which contains all sequences of any HCDR3 length; (2) long HCDR3s, which contains only sequences with a HCDR3 length ≥24 amino acids; and (3) very long HCDR3s, which contains only sequences with a HCDR3 length ≥28 amino acids. The frequency of each germline variable gene family, diversity gene family, and joining gene was determined for each HCDR3 length group. The mean frequency ± SEM is shown. All HCDR3 lengths were calculated using the IMGT numbering system. The p values were determined using a two-way ANOVA with Bonferroni post-tests. All statistically significant differences are indicated. * = p<0.05, ** = p<0.01, *** = p<0.001. (TIF) [file pone.0036750.s002.tif]

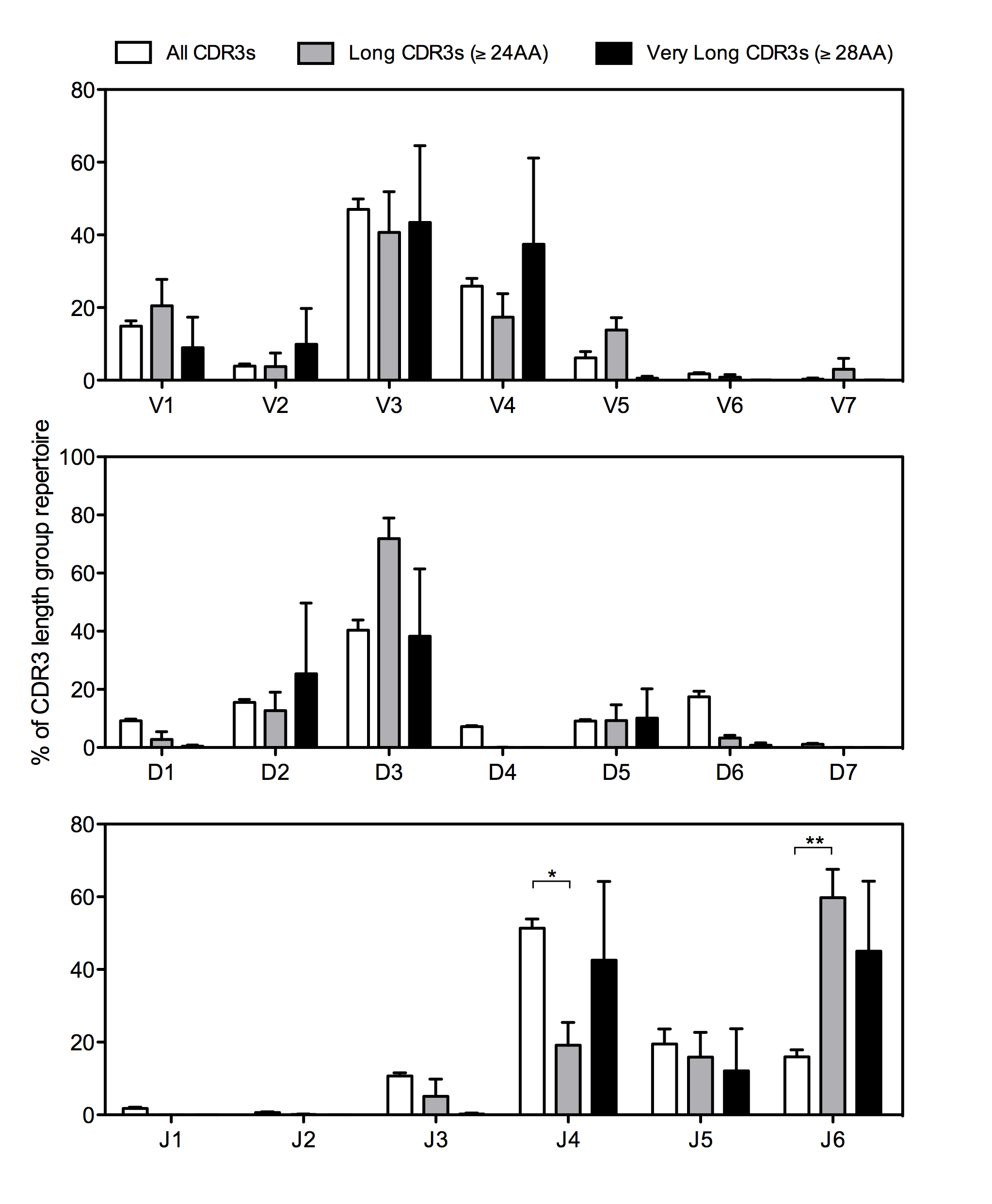

Supplement: Figure S3 — Skewed germline gene usage in IgG memory antibodies containing long or very long HCDR3s. Peripheral blood antibody sequences from the IgG memory subset of Group 1 (n = 4) healthy donors were assembled into the following three groups by HCDR3 length: (1) all HCDR3s, which contains all sequences of any HCDR3 length; (2) long HCDR3s, which contains only sequences with a HCDR3 length ≥24 amino acids; and (3) very long HCDR3s, which contains only sequences with a HCDR3 length ≥28 amino acids. The frequency of each germline variable gene family, diversity gene family, and joining gene was determined for each HCDR3 length group. The mean frequency ± SEM is shown. All HCDR3 lengths were calculated using the IMGT numbering system. The p values were determined using a two-way ANOVA with Bonferroni post-tests. All statistically significant differences are indicated. * = p<0.05, ** = p<0.01, *** = p<0.001. (TIF) [file pone.0036750.s003.tif]

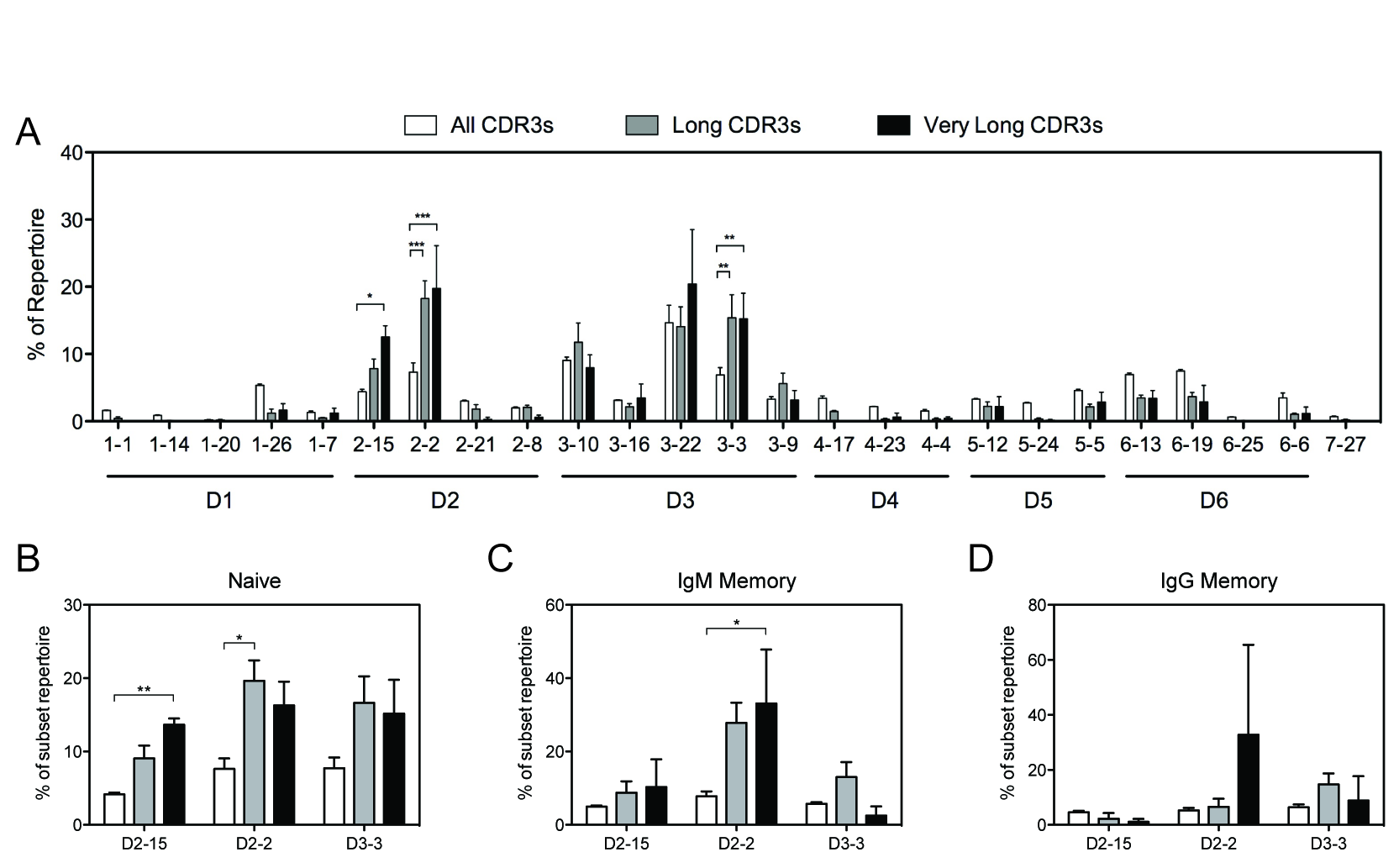

Supplement: Figure S4 — Frequency of D gene use in long and very long HCDR3s. Antibody sequences from Group 1 healthy donors (n = 4) were assembled into the following three groups by HCDR3 length: (1) all HCDR3s, which contains all sequences of any HCDR3 length; (2) long HCDR3s, which contains only sequences with a HCDR3 length ≥24 amino acids (at least two standard deviations above the mean HCDR3 length for the entire repertoire); or (3) very long HCDR3s, which contains only sequences with a HCDR3 length ≥28 amino acids (at least three standard deviations above the mean). The frequency of diversity gene use in each of the three groups was determined for Group 1 healthy donor sequences (A). For the diversity genes that showed significant increases in (A), frequency was calculated for the naive (B), IgM memory (C), and IgG memory (D) subsets from the same Group 1 donors. The mean frequency ± SEM is shown. All HCDR3 lengths were calculated using the IMGT numbering system. The p values were determined using a two-way ANOVA with Bonferroni post tests. All statistically significant differences are indicated. * = p<0.05, ** = p<0.01, *** = p<0.001. (TIF) [file pone.0036750.s004.tif]

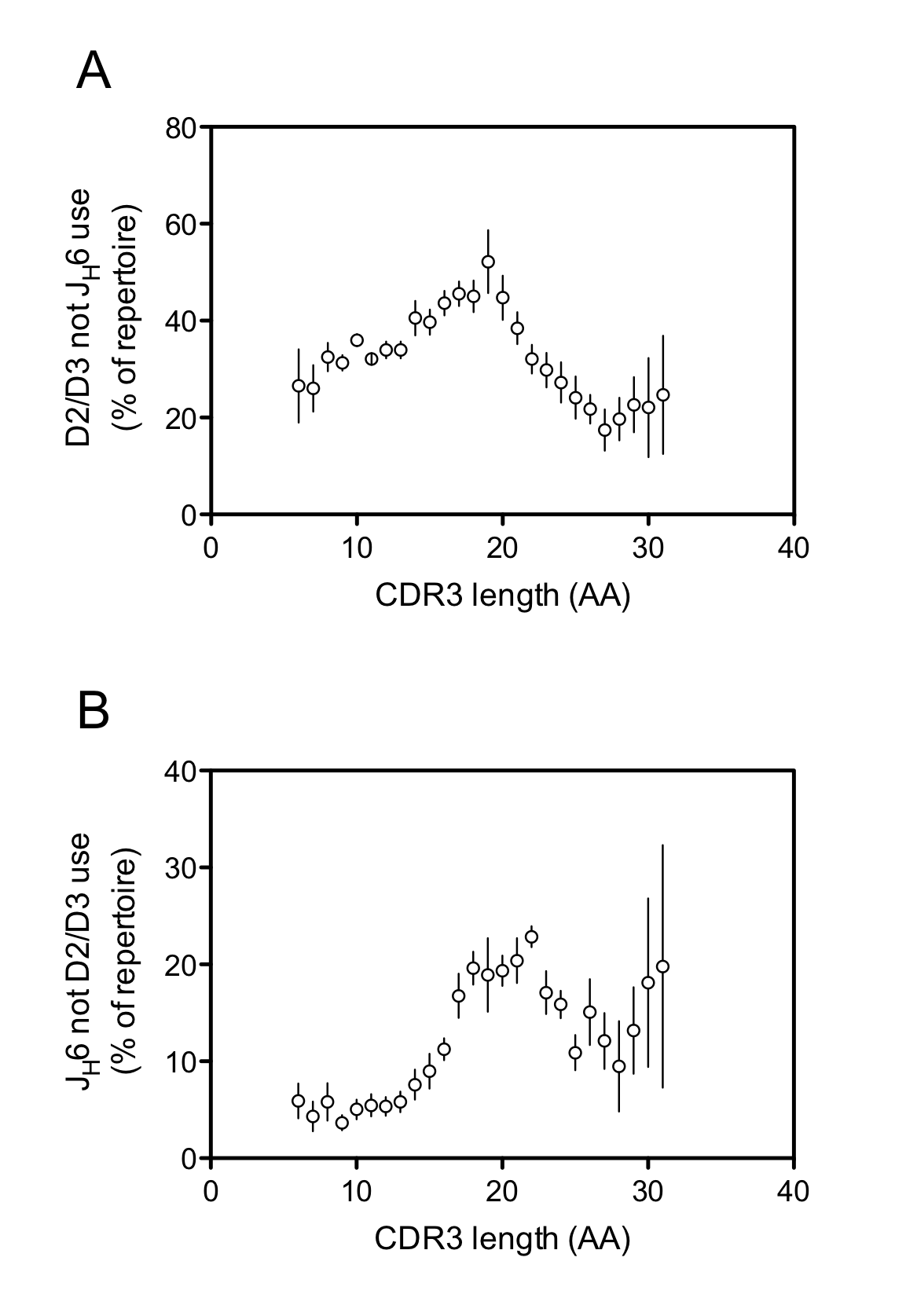

Supplement: Figure S5 — Limited preference for isolated use of D2/D3 or JH6 in the longest HCDR3s. Peripheral blood antibody sequences from Group 1 (n = 4) and Group 2 (n = 3) healthy donors were grouped by HCDR3 length (in amino acids) and the frequency of (A) sequences encoding germline genes from the D2 or D3 families but not JH6 or (B) sequences encoding JH6 but not either of the D2 or D3 families. The mean frequency ± SEM is shown. All HCDR3 lengths were calculated using the IMGT numbering system. (TIF) [file pone.0036750.s005.tif]
