# Supplementary material for: Human Peripheral Blood Antibodies with Long HCDR3s Are Established Primarily at Original Recombination Using a Limited Subset of Germline Genes
Source: PLoS One. 2012 May 9;7(5):e36750. doi: 10.1371/journal.pone.0036750 (PMC3348910; doi:10.1371/journal.pone.0036750)
Supplement: Table S1 — Primers used in RT-PCR and 454-Adapter PCR. Multiplex identifiers (MIDs) are underlined. Forward primers use MID7 and reverse primers use MID4 (Roche). Additional complementarity between RT-PCR primers and 454-Adapter PCR primers is identified in italics. All primer sequences are in the 5′ to 3′ orientation. (DOCX) [file pone.0036750.s006.docx]

**Table S1: Primers used in RT-PCR and 454-Adapter PCR.** Multiplex identifiers (MIDs) are underlined. Forward primers use MID7 and reverse primers use MID4 (Roche). Additional complementarity between RT-PCR primers and 454-Adapter PCR primers is identified in italics. All primer sequences are in the 5’ to 3’ orientation.

**PRIMER NAME SEQUENCE TYPE**

VH1/7-FR1 *CCATCAG*CGTGTCTCTAGGCCTCAGTGAAGGTCTCCTGCAAG RT-PCR

VH2-FR1 *CCATCAG*CGTGTCTCTAGTCTGGTCCTACGCTGGTGAACCC RT-PCR

VH3-FR1 *CCATCAG*CGTGTCTCTACTGGGGGGTCCCTGAGACTCTCCTG RT-PCR

VH4-FR1 *CCATCAG*CGTGTCTCTACTTCGGAGACCCTGTCCCTCACCTG RT-PCR

VH5-FR1 *CCATCAG*CGTGTCTCTACGGGGAGTCTCTGAAGATCTCCTGT RT-PCR

VH6-FR1 *CCATCAG*CGTGTCTCTATCGCAGACCCTCTCACTCACCTGTG RT-PCR

JH Consensus *CGCTCAG*AGCACTGTAGCTTACCTGAGGAGACGGTGACC RT-PCR

454-Adapter-A CGTATCGCCTCCCTCGCG*CCATCAG*CGTGTCTCTA 454 PCR

454-Adapter-B CTATGCGCCTTGCCAGCC*CGCTCAG*AGCACTGTAG 454 PCR
