# Supplementary material for: Human Peripheral Blood Antibodies with Long HCDR3s Are Established Primarily at Original Recombination Using a Limited Subset of Germline Genes
Source: PLoS One. 2012 May 9;7(5):e36750. doi: 10.1371/journal.pone.0036750 (PMC3348910; doi:10.1371/journal.pone.0036750)
Supplement: Table S2 — Sequencing results from Group 2 healthy donors and HIV-infected donors. Donor IDs with the format 100XX are HIV-infected donors. Donors with the format HD-XXX and Subject 42 are Group 2 healthy donors. The number of sequences includes only non-redundant, high-quality sequences. (DOCX) [file pone.0036750.s007.docx]

**Table S2. Sequencing results from Group 2 healthy donors and HIV-infected donors.** Donor IDs with the format 100XX are HIV-infected donors. Donors with the format HD-XXX and Subject 42 are Group 2 healthy donors. The number of sequences includes only non-redundant, high-quality sequences.

**DONOR ID SEQUENCES**

10002 388,714

10028 351,722

10042 110,431

10076 333,738

HD-184 127,583

HD-229 70,168

Subject 42 102,846
